# Combined trauma in craniomaxillofacial and orthopedic-traumatological patients: the need for proper interdisciplinary care in trauma units

**DOI:** 10.1007/s00068-020-01479-x

**Published:** 2020-08-31

**Authors:** Nils Mühlenfeld, Philipp Thoenissen, René Verboket, Robert Sader, Ingo Marzi, Shahram Ghanaati

**Affiliations:** 1 Department of Trauma, Hand and Reconstructive Surgery, Goethe University Frankfurt, Theodor-Stern-Kai 7, Frankfurt am Main, 60590 Germany; 2 Department of Oral, Cranio-Maxillofacial and Plastic Facial Surgery, Goethe University Frankfurt, Theodor-Stern-Kai 7, 60590 Frankfurt am Main, Germany

**Keywords:** Craniomaxillofacial injuries, Spine fractures, Brain injuries, Emergency treatment

## Abstract

**Aim:**

The primary aim of this study was to analyze frequency and characteristics of combined facial and peripheral trauma with consecutive hospitalization and treatment.

**Materials and methods:**

The study included all patients with concomitant orthopedic-traumatolgical (OT) and craniomaxillofacial (CMF) injuries admitted to our level I trauma center in 2018. The data were collected by analysis of the institution’s database and radiological reviews and included age, sex, injury type, weekday and time of presentation. All patients were examined and treated by a team of surgeons specialized in OT and CMF directly after presentation.

**Results:**

A total number of 1040 combined OT and CMF patients were identified. Mean age was 33.0 ± 26.2 years. 67.3% (*n* = 700) were male patients. Primary presentation happened most frequently on Sundays (*n* = 199) and between 7 and 8 pm (*n* = 74). 193 OT fractures were documented, where cervical spine injuries were most frequent (*n* = 30). 365 facial and skull fractures were recorded. 10.8% of the 204 patients with fractures of the viscerocranium presented with at least one fracture of the extremity, 7.8% (16/204) with cervical spine fractures, 33.3% (68/204) with signs of closed brain trauma and 9.8% (20/204) with intracranial hemorrhage.

**Discussion:**

The study shows a high frequency of combined facial with OT-injuries and brain damage in a predominantly young and male cohort. Attendance by interdisciplinary teams of both CMF and OT surgeons specialized in cervical spine trauma surgery is highly advisable for adequate treatment.

**Conclusion:**

Diagnostics and treatment should be performed by a highly specialized OT and CMF team, with a consulting neurosurgeon in a level-1 trauma center to avoid missed diagnoses and keep mortality low.

## Introduction

Combined craniomaxillofacial (CMF) and orthopedic-traumatological (OT) injuries are common and pose a challenge to traumatological teams in the emergency unit. These injuries predominantly result from assaults and high energy traumata like traffic accidents [1–5].

Extremity and spinal fractures often need to be stabilized and/or reconstructed within the first few hours after trauma. Some CMF diagnoses such as retrobulbar hematoma or multifragmentary fractures of the jaw demand immediate surgery such as orbital decompression and or airway preservation.

In contrast, patients with concomitant CMF and OT injuries have a high mortality on the trauma site and in the hospital, as well as a high risk to be misdiagnosed [6, 7].

Yet, only few emergency units are in a position to provide immediate treatment by an interdisciplinary team of CMF surgeons and OT surgeons with a consulting neurosurgeon at all times.

While some data are available from small-scale studies concerning the combination of facial trauma with brain injuries as well as thoracic trauma [1, 8, 9], little data are presented about frequency and extent of concomitant CMF and OT-injuries.

The primary aim of this study was to analyze frequency and characteristics of combined facial and peripheral trauma as well as the consecutive hospitalization and management. In addition, the importance of an interdisciplinary team consisting of CMF as well as OT surgeons in an emergency unit is discussed.

This study was conducted at the university hospital Frankfurt which is a tertiary hospital and national trauma center with a large combined rural and metropolitan catchment area and provides specialized and interdisciplinary medical care for over 320.000 patients per year and a draining catchment population of 3–4 million people. The university hospital Frankfurt maintains a central emergency unit with doctors of the internal medicine and surgical professions being present at all times. This includes the availability of representatives of the Department of Trauma, Hand and Reconstructive Surgery, as well as of the Department of Oral, Cranio-Maxillofacial and Plastic Surgery for 24 hours, 7 days a week.

## Materials and methods

### Data acquisition

Approval from the institutional review board of the medical faculty (20–532) was obtained prior to performing this retrospective study. The study included all 1040 patients with concomitant facial and traumatological injuries who were admitted to our level I trauma center in 2018. Patients were identified via a retrospective systematical query in the Hospital Information System (HIS) using the International Statistical Classification of Diseases and Related Health Problems Version 10 (ICD-10) codes for traumatological and CMF diagnoses of the German Diagnosis Related Groups (G-DRG). The data were collected by analysis of the institution’s data-base, charts, and radiological reviews. All patients were double-checked in view of G-DRG code and clinical information. Unclear or falsely coded patients were strictly excluded from the analysis. Admission information obtained included age, sex, injury type as well as the weekday and time of presentation. All patients were treated by a team of surgeons specialized in orthopedic trauma care and CMF-surgeons directly after presentation. OT surgeons primarily assessed and managed acute body cavity trauma (chest, abdomen and pelvis), with an abdominal surgeon for consultation as a backup. Patients underwent standard of care imaging that included FAST-sonography of the abdomen, radiographs including computerised tomography imaging (CT of skull, midface including air sinuses, maxilla and mandible in axial, coronal and sagittal planes with slice thickness of 1 mm and three-dimensional (3D) reformats as appropriate). In case of a detected intracranial injury, a neurosurgeon was then directly consulted. Necessary emergency craniotomy or drill trepanation was performed by surgeons specialized in neurosurgery. Patients that had massive brain trauma as leading injury were excluded from this study, as they were allocated for neurosurgery treatment at emergency site and treated in the department of neurosurgery only.

### Statistical analysis

Statistical analysis was performed using Excel and Prism Graphpad using a non-parametric Mann–Whitney-Test. *P* values < 0.05 were considered to be significant. The diagnoses were collected and descriptively and exploratively characterized and analyzed. Next to purely descriptive methods, time series statistics and periodical day influences were analyzed.

## Results

### Characteristics of patient’s collective

A total number of 1040 of combined OT and CMF patients were identified. Mean age was 33.0 ± 26.2 years (1;99 years). 32.7% (*n* = 340) were female, 67.3% (*n* = 700) were male patients.

### Characteristics of hospitalization: high demand outside of day-routine hours

All patients presenting to the emergency unit were differentiated into OT and CMF patients

**Fig. 1:**
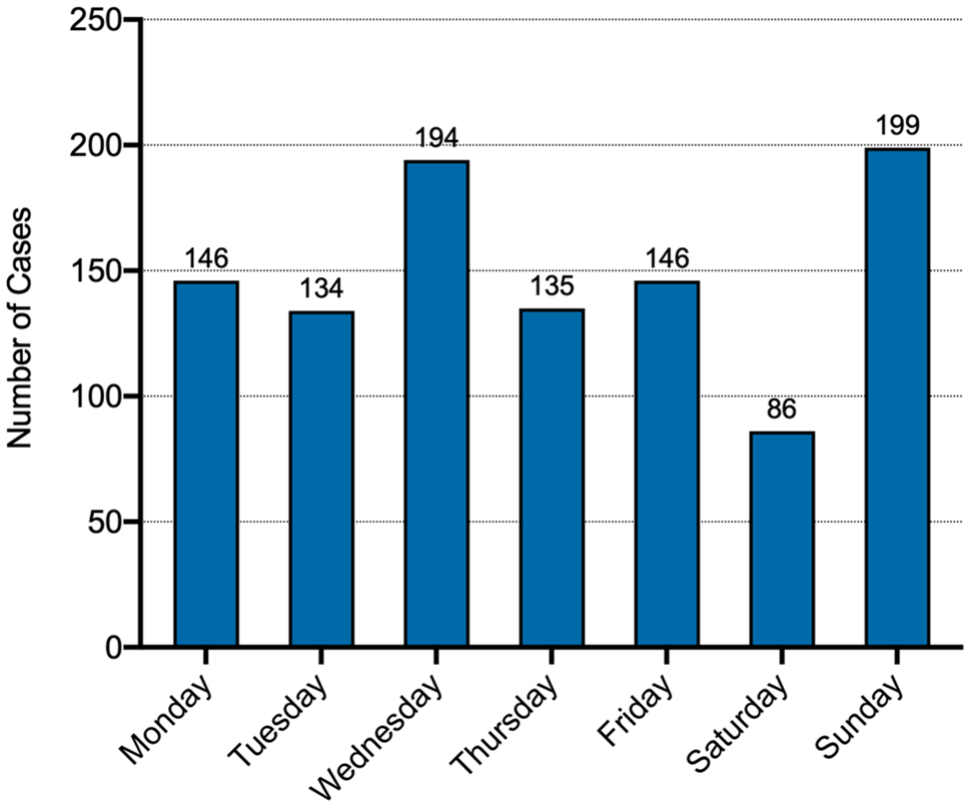
Total number of patients admitted per day in 2018: most presentations were noted for Sunday (*n* = 199), Wednesday (*n* = 194), followed by Monday and Friday (*n* = 146)

**Fig. 2:**
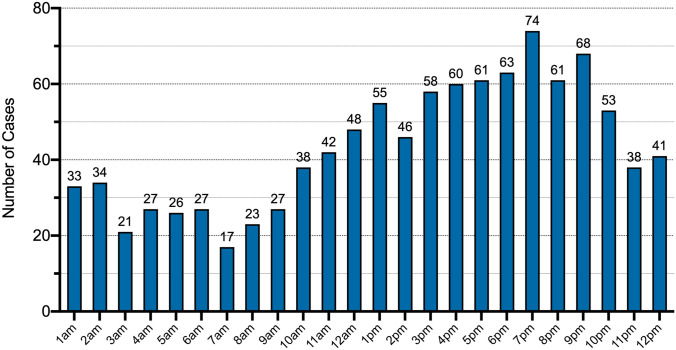
Total number of patients presenting per hour in 2018: presentations start at 7 am and reach a top at 7 pm, with most presentations between 7 and 8 pm over day period

Primary presentation happened most frequently on Sundays (*n* = 199) and on Wednesdays (*n* = 194). Patients presented most frequently between 7 and 8 pm local time (*n* = 74). The majority of all patients in 2018 presented in our emergency unit outside the regular work hours (*n* = 627, 60.3%) (Figs. 1 and 2).

Of 1040 patients in our cohort a total of 273 patients (26.3%) became inpatients and 767 (74.7%) were discharged the same day after emergency treatment.

All patients were viewed and treated by both departments in close cooperation.

Patients presenting with minor injuries (i.e., headwounds, contusions, simple fractures) and no indication for immediate inpatient treatment were planned for further outpatient care or discharged into the ambulatory sector.

### Distribution and treatment of OT injuries

193 OT fractures were documented in our cohort (top three categories: cervical-spine fractures 30, combined fractures of the wrist, hand and fingers 34, and rib fractures 27).

In 88 patients (8.5% of the whole cohort), immediate (*n* = 32; 36.4%) or post-primary (*n* = 56; 63.6%) surgery had to be performed by a specialized OT surgeon. Closed reductions of 14 fractures were performed temporarily by external fixation. 15 cervical spines were stabilized by either plates or screws and 6 dorsal stabilizations of the thoracolumbar spine were performed. Eight femurs and six tibias were nailed intramedullary. One femur required total hip arthroplasty. 30 bones in upper arm, the forearm and the hand, as well as 12 clavicles one tibia and one femur were reduced with locking plates.

Out of 17 pneumothoraces and 2 hemothoraces, 12 had to be treated with Bulau’s drainage as an emergency procedure within our emergency unit. Also 8 lung contusions were documented (Fig. 1). Others were simple injuries such as cuts, bruises or contusions.

### Distribution of CMF injuries

**Fig. 3:**
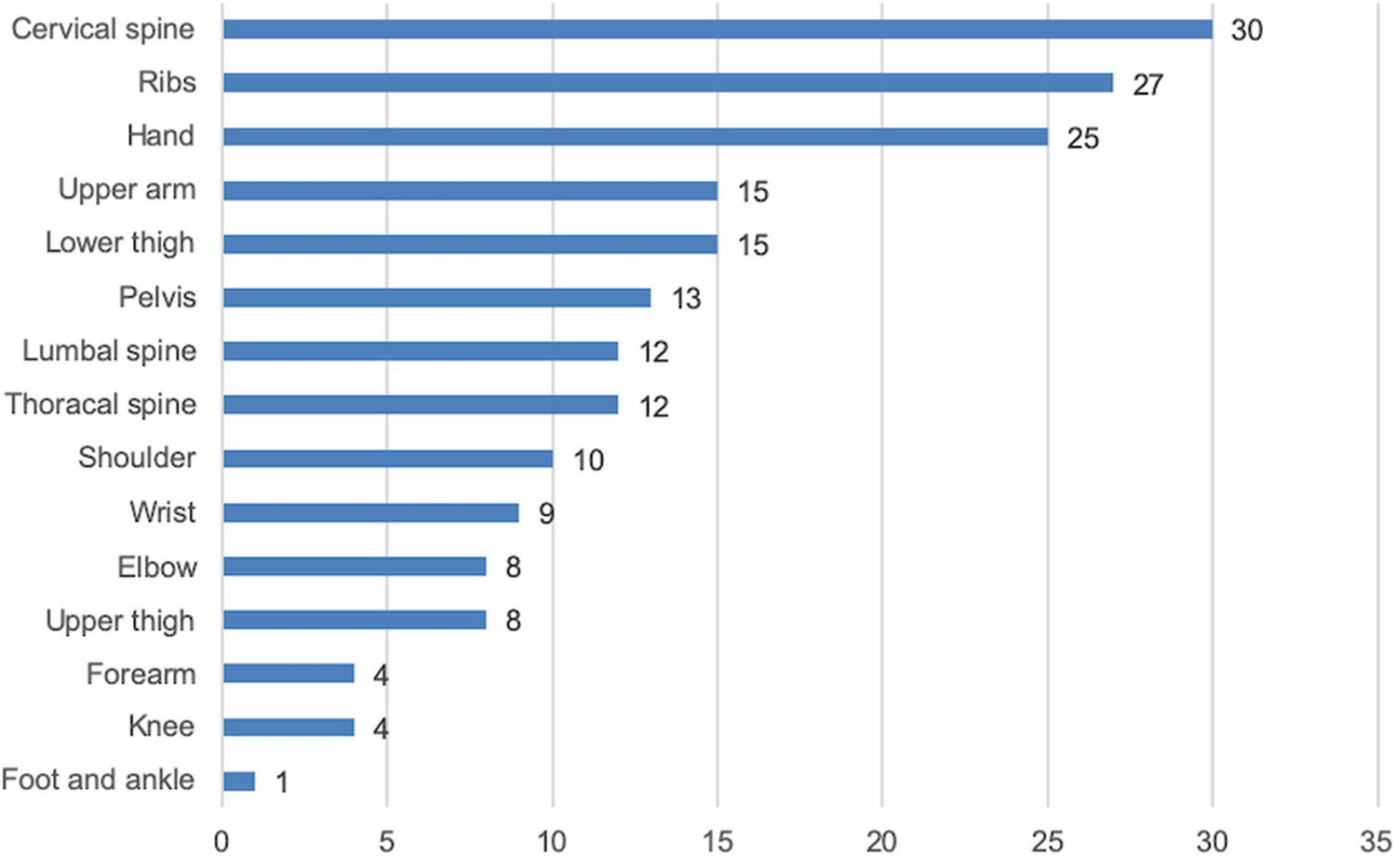
Distribution of orthopedic-trauma-(OT)-fractures in our cohort: cervical spine fractures were most frequent (*n* = 30) followed by rib fractures (27) and hands (25)

365 facial and skull fractures were recorded (top three categories: fractures to the nose 119, orbital floor and roof fractures 62, centro-lateral-midface fractures 56 (isolated one or two side centro-lateral midface-fractures including fractures of maxillary and zygomatic bone). 19.6% (*n* = 204) of the patients presented with at least one fracture of the viscerocranium, i.e., fracture to the nose, centro-lateral midface fractures, fracture of maxilla, orbital floor or roof and zygomatic bone). 46 patients had at least one fractured tooth, and 7 at least one dislocated tooth. Others were simple injuries such as cuts, bruises or contusions (Fig. 3).

**Fig. 4:**
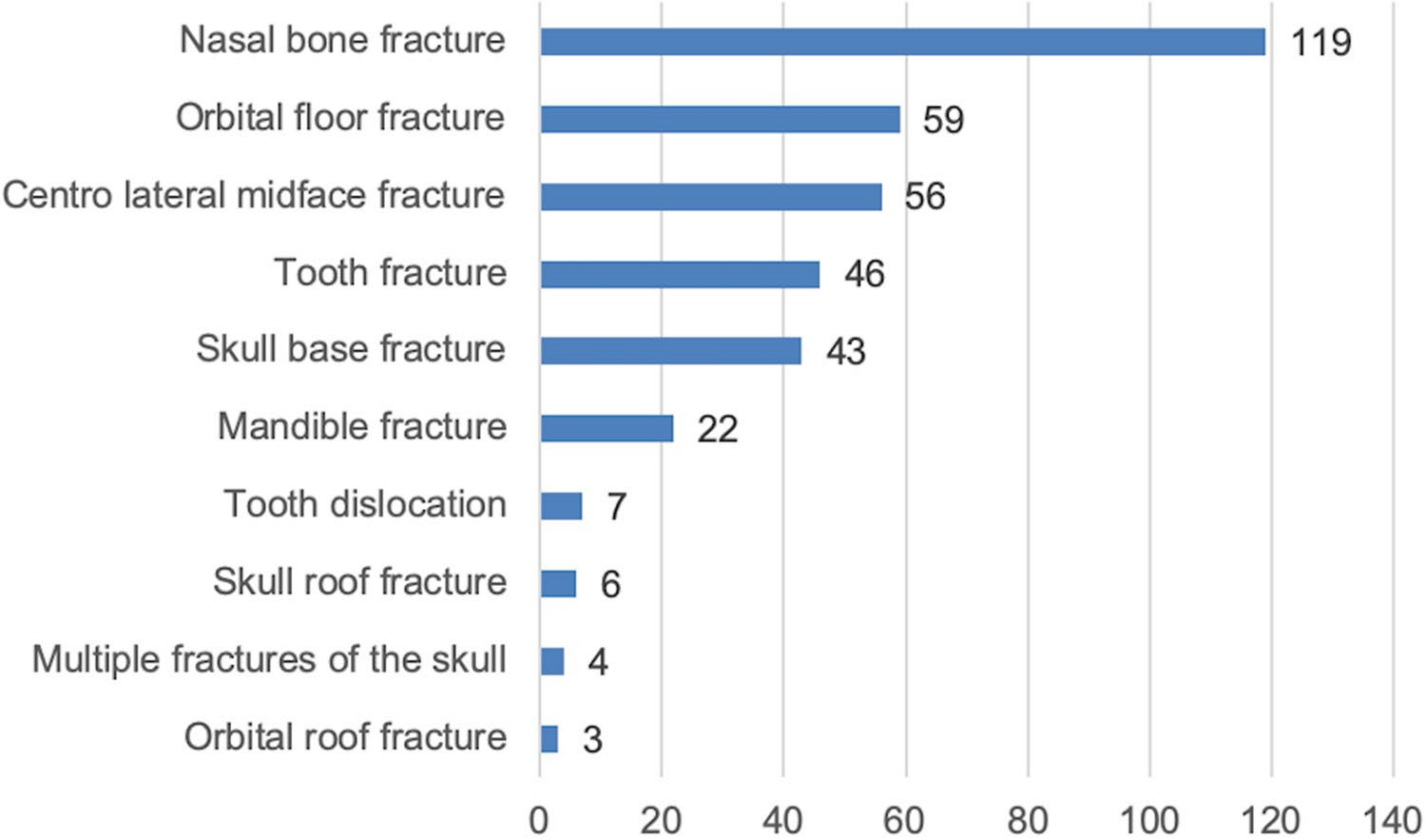
Distribution of craniomaxillofacial-(CMF)-injuries in our cohort: nasal bone fractures were most frequent with *n* = 119 followed by orbital floor fractures (59), zygomatic bone fractures (56)

Nasal bones were repositioned 41 times. Zygomatic bones were reconstructed in 29, orbital bones in 24 and mandibles in 21 patients. Bilateral midface-fracture-reconstructions were performed in 19 patients. Seven tracheotomies were performed as emergency measures (Fig. 4).

### Injuries to the brain

**Fig. 5:**
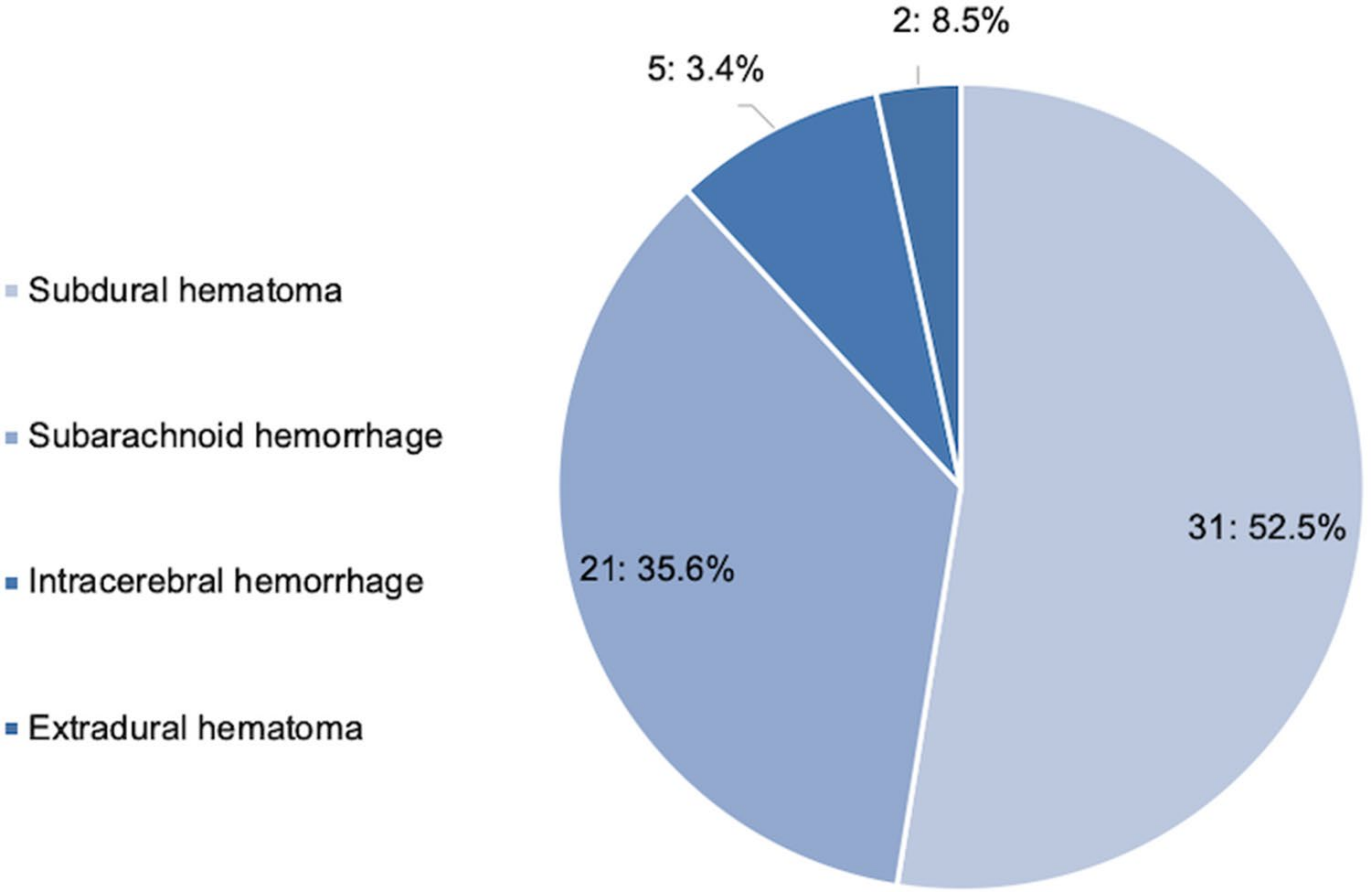
Types of brain-injuries in our cohort: subdural hematoma (*n* = 31), subarachnoid hemorrhage (21), intracerebral hemorrhage (5), extradural hematoma (2)

Aside from the head wounds (*n* = 640), craniocerebral trauma was by far the most documented injury in our cohort (21.8%, *n* = 227). 168 patients (16.2% of all) had signs of trauma to the brain like repeated vomiting, amnesia and/or unconsciousness without any correlation to pathological findings in the CT-scan of the brain (76%). 59 CT-scan proven intracranial hemorrhages were documented with subdural hematoma occurring predominantly with 52.5% (*n* = 31). 35.6% (*n* = 21) were documented subarachnoid hemorrhages, 8.5% (n = 2) extradural hematomas and 3.4% (*n* = 5) intracerebral hemorrhages (Fig. 5).

Only 13 patients out of our cohort required emergency neurosurgical treatment (1.3%) during their stay in 2018. An emergency craniotomy was performed in ten and a drill trepanation in three patients to prevent irreparable brain damage.

Patients presenting with fractures of the viscerocranium showed a higher association with concomitant closed brain-damage (52.1%) than with extremity fractures (10.8%).

### Concomitant OT and CMF fractures

Of the 1040 patients with combined OT and CMF-injuries, 15.1% (*n* = 157) presented with at least three, and 2.5% (*n* = 26) with at least four documented injuries after trauma.

The top three combinations of OT fractures with CMF fractures were:

Fractures of the nose combined with cervical spine fractures (*n* = 14), fractures of the nose combined with serial rib-fractures (*n* = 9) and fractures of the wrist, hand and fingers combined with fractures of the orbita *n* = 9.

Overall, the prevalence of midface fractures combined with cervical spine injuries was 7.8% (16/204).

Thirty-one patients (3.0% of our cohort) had to receive surgical treatment by a CMF as well as an OT surgeon. Only three patients had to be treated by a CMF, an OT and a neurosurgeon.

In addition, the majority of concomitant trauma were minor injuries. Open wounds of the head and extremities were documented in 650 patients, which were seen in our emergency unit by doctors of both specialties, with the facial wounds being repaired by the CMF surgeons.

### Mortality

Of the 1040 patients with combined OT and CMF diagnoses, nine died in our hospital (0.9%), of which five had an ISS Injury Severity Score of 75. The Score (ISS) of the in-hospital deaths was 61.9 ± 18.3. The most frequent cause of death was massive intracranial hemorrhage with cerebral contusion (55.6%, 5/9). Three of these patients died in the shock room shortly after presentation. Four other patients of those died of varying causes: one because of unstoppable bleeds in the thorax, and one of hospital-acquired pneumonia. Cardiac arrest prior to hospitalization despite attempted re-animation/rescucitation was the cause of death in two patients.

## Discussion

Our results show a high frequency of combined injuries to the face along with OT-injuries, as well as brain damage (*n* = 1040, 33.0 ± 26.2 years old) in a predominantly young and male cohort (*n* = 700; 67.3% male).

A large proportion of our cohort (15.5%) presented with at least three injuries (2.5% with four injuries) that were documented. Patients with combined OT and CMF traumas, therefore, require a differentiated and thorough diagnostic approach to treatment.

The severity of trauma in patients with combined OT and CMF injuries manifests itself also in the high ratio of needed inpatient treatment. In our cohort, 26.3% required treatment as inpatients (273/1040).

Thirty-one patients (3.0% of our 2018 cohort) had to receive surgical treatment by both, a CMF as well as an OT surgeon during their inpatient treatment after trauma. Only three patients required combined treatment by a CMF, an OT, as well as a neurosurgeon within the first hours after presentation. These are new findings, as little data for teamwork treatment of OT-surgeons, CMF-surgeons and neurosurgeons exist.

According to the time of first visit of the patients and trauma patterns the presence of the required specialists’ teams can only be ensured by larger trauma centers with a CMF department in the building that has critical mass and sufficient personnel to provide full treatment also at nights and on weekends.

### Extremity fractures in CMF trauma patients

8.7% of all our 1040 patients, and 10.8% of the 204 patients with midface fractures presented with at least one fracture of the extremity. This rate is noticeably lower than the incidence of 33% as previously reported by Carlin et Al. [2]. The leading cause for midface fractures has shown to be high energy trauma like car accidents or falls from great heights [3]. Our low rate of extremities may be due to the vast enhancement in safety within cars, as improved airbags and crumple zones contribute to prevent broken extremity bones [10, 11].

### Brain trauma in combined CMF- and OT-patients

We noticed a high incidence of brain trauma with facial trauma in our cohort (18.2%). This matches well with small-level data from other papers reporting a close relation between severe brain trauma as evidenced by epidural and subdural hemorraghes, and brain contusion in association with midface fractures [1, 8, 9]. Already in 1998, a high association of closed brain trauma with unconsciousness (40%) in patients with midface fractures was observed by Carlin et al. in a 10-year retrospective study [2]. In our cohort of patients with midface fractures, 33.3% presented with signs of closed brain trauma combined with unconsciousness, repeated vomiting or amnesia (68/204). This seems to be more frequent compared to the occurrence of extremity fractures in patients with midface fractures as they are represented with 10.8%. 9.9% showed intracranial hemorrhage as detected by either CT or MRI (20/204). Fractures of the skull base were highly associated with intracranial hemorrhages. In 46.5% of the detected skull base fractures, intracranial hemorrhage was also detected. In view of the high incidence of brain trauma, radiographed diagnostics of the intracranial structures is recommended as generic approach when patients present with signs of craniocerebral trauma. At least, inpatient surveillance and/or monitoring should be applied [12].

These injury patterns also show the severity of patients with combined OT and CMF injuries. A patient suspect of having a combined trauma should, therefore, always be checked by specialists of both disciplines, with available neurosurgical consultation as soon as possible. This can be a challenge due to the known large proportion of intoxicated patients [13–19].

With the majority of the cohort demanding immediate treatment outside of the usual clinic-hours, surgeons specialized in CMF need to be available in the hospital for 24 h, 7 days a week.

### Cervical spine injuries in patients with midface fractures

There is a well-documented increased risk for injuries of the cervical spine in trauma patients that suffer midface fractures. Midface fractures propose an independent risk factor for cervical spine injuries [20]. In our cohort the incidence of cervical spine fractures in patients with midface fractures was 7.8% (16/204) which indicates similar relevant levels as previously reported by other teams (2.6% by Ardekian et al.—9.7% by Mithani et al*.*) [20–25]. Therefore, diagnostics and treatment of CMF and OT patients in trauma centers with a department for spinal surgery is strongly advised.

### Low mortality rate in specialized centers

In our cohort, the mortality rate presented low with death occurring in 0.9% after trauma (ISS 61.9 ± 18.3). This shows that with a large caseload over 1000 patients per year and treatment in centers with a high level of specialty of both departments being present in the emergency room, a significant better outcome is reached. Unfortunately, this is still only the case in few emergency units. Preclinical triage is needed to address the right trauma center for the patient. A quick transport to a large trauma center where both departments are present at all times should be advised when preclinically a combined injury is presumed.

### Limitation of the study

In our cohort we only investigated data from one trauma center with both departments present. For a bigger collection of data, multicenter-studies over the course of a couple of years are necessary. Still, the large number of patients represented by our evaluation provide a good basis for further studies.

## Conclusion

Patients with relevant combined OT and CMF injuries are frequently admitted to the emergency unit. A large proportion need specialized surgical treatment of either or both specialties. A neurosurgeon should be available, at least in a consulting function at all times. The remaining high mortality may be caused by the often-delayed treatment by doctors that are adequately specialized within hospitals that do not have a department of CMF surgery. In our cohort the mortality after presentation was relatively low with 1.0% percent (*n* = 10 of 1040). With a caseload of 1040 presentations in a year, a trauma center that provides a team of OT surgeons who treat spinal injuries on a regular basis, as well as highly specialized CMF surgeons, is needed to ensure an adequate and immediate treatment to reduce mortality and complications. Since a majority of the patients presented on the weekends and at night, the team should be available at all times and, therefore, present in the hospital.
